# Malic Enzyme 1 (ME1) is pro-oncogenic in Apc^Min/+^ mice

**DOI:** 10.1038/s41598-018-32532-w

**Published:** 2018-09-24

**Authors:** Lorenzo M. Fernandes, Ahmed Al-Dwairi, Rosalia C. M. Simmen, Meera Marji, Dustin M. Brown, Sarah W. Jewell, Frank A. Simmen

**Affiliations:** 1 Interdisciplinary Biomedical Sciences Program, University of Arkansas for Medical Sciences, Little Rock, AR 72205 USA; 2 Department of Physiology & Biophysics, University of Arkansas for Medical Sciences, Little Rock, AR 72205 USA; 3 The Winthrop P. Rockefeller Cancer Institute, University of Arkansas for Medical Sciences, Little Rock, AR 72205 USA

## Abstract

Cytosolic Malic Enzyme (ME1) provides reduced NADP for anabolism and maintenance of redox status. To examine the role of ME1 in tumor genesis of the gastrointestinal tract, we crossed mice having augmented intestinal epithelial expression of ME1 (ME1-Tg mice) with Apc^Min/+^ mice to obtain male Apc^Min/+^/ME1-Tg mice. ME1 protein levels were significantly greater within gut epithelium and adenomas of male Apc^Min/+^/ME1-Tg than Apc^Min/+^ mice. Male Apc^Min/+^/ME1-Tg mice had larger and greater numbers of adenomas in the small intestine (jejunum and ileum) than male Apc^Min/+^ mice. Male Apc^Min/+^/ME1-Tg mice exhibited greater small intestine crypt depth and villus length in non-adenoma regions, correspondent with increased KLF9 protein abundance in crypts and *lamina propria*. Small intestines of male Apc^Min/+^/ME1-Tg mice also had enhanced levels of *Sp5* mRNA, suggesting Wnt/β-catenin pathway activation. A small molecule inhibitor of ME1 suppressed growth of human CRC cells *in vitro*, but had little effect on normal rat intestinal epithelial cells. Targeting of ME1 may add to the armentarium of therapies for cancers of the gastrointestinal tract.

## Introduction

Metabolism underpins virtually all aspects of cellular functioning. The complex interplay (and regulation thereof) between catabolic and anabolic pathways for nucleic acids, carbohydrates, sterols, lipids and proteins serves to maintain cellular homeostasis, as well as support the shift from resting to proliferative state and back. It is, therefore, not surprising that specific perturbations in cellular metabolism are linked to the initiation and progression of multiple cancers, including those of the colorectum (colorectal cancers (CRC))^1–5^. Cancer cells depend on a copious supply of energy as well as efficient biosynthetic machinery for cell division, and often reprogram metabolic pathways to fulfill these requirements^5,6^. It follows that the identification of genes and proteins that cancer cells harness to fulfill their heightened metabolic requirements may yield new strategies to prevent and/or reduce tumor initiation and growth as well as metastasis.

Malic Enzymes (ME) participate in reactions that link anabolic and catabolic branches of metabolism^6–9^. Malic enzymes are involved in the oxidative-decarboxylation of malate to pyruvate using NAD or NADP as required co-factor to generate NADH or NADPH, respectively^7^. Several forms of malic enzyme are found in mammals, the cytosolic ME which is designated Malic Enzyme 1 (ME1) and the mitochondrial forms which include Malic Enzyme 2 (ME2) and Malic Enzyme 3 (ME3). ME1 and ME3 require NADP for catalytic activity whereas ME2 utilizes either NAD or NADP. Perturbations in ME abundance and/or activity have been demonstrated in multiple cancers and cancer cell lines^6,8–11^. Due to its cytoplasmic localization, ME1 links the catabolic pathways of glycolysis and the Krebs cycle (via the conversion of malate to pyruvate) to the anabolic pathways of fatty acid and cholesterol biosynthesis through NADPH. Increased *de novo* fatty acid and cholesterol biosynthesis are evident in multiple tumor types and are thought to drive, in part, their proliferative and anti-apoptosis phenotypes^8–13^. Moreover, cancer cells employ lipids for a wide range of processes including the production of new membranes, intracellular trafficking, cell signaling, and as an energy source during periods of nutrient deprivation. ME1 contributes to the cytoplasmic pool of NADPH, a fraction of which is used by Fatty Acid Synthase (FASN), a primary lipogenic enzyme that is upregulated in many cancers and is itself implicated in tumor genesis and metastasis^12–14^. ME1 also participates in the maintainance of the redox balance in cells and its cytoplasmic localization may facilitate its functional and physical interactions with other proteins in novel pathways.

Several lines of evidence link ME1 to regulation of cancer cell growth. Tumor protein 53 (TP53) was shown to suppress ME1 and to induce cell senescence^15^. Further, CRC cells harboring an oncogenic mutant form of the KRAS gene had enhanced expression of ME1^16^. Mutations of both TP53 and KRAS are common in CRC^17^. Indeed, siRNA-mediated knockdown of ME1 leads to growth inhibition and senescence of CRC cell lines *in vitro*^9,15^. Moreover, ME1 abundance in some cancer cells was reported to be a prognostic marker for efficacy of radiation therapy^18^. Nevertheless, most of what is known about the actions of ME1 in cancer cells is derived from *in vitro* studies and xenograft transplants to mice.

To mechanistically define the contributions of ME1 to intestinal cancer genesis within a more physiological context, we generated an ME1 transgenic mouse (ME1-Tg) which over-expresses ME1 predominantly in intestinal epithelial cells under the control of the murine villin gene promoter-enhancer^19^. We reported that ME1-Tg mice had greater intestinal 5-bromodeoxyuridine labeling index and exhibited deeper intestinal and colonic crypts^19^. In contrast, a functionally null ME1 mouse (MOD-1 mouse line) displayed shallower colonic crypts and reduced intestinal expression of pro-proliferative *Ccnd1* and *Mtor* genes compared to WT mice^20^. Intestinal expression of genes encoding proteins responsible for lipid and cholesterol biosynthesis were elevated in the ME1-Tg mice^19^, indicating a shift towards increased lipogenesis. While these studies suggested a stimulatory role for ME1 in proliferation of gut epithelium, ME1-Tg mice did not spontaneously develop intestinal adenomas at increased frequencies.

The Apc^Min/+^ mouse is a well-utilized model of Familial Adenomatous Polyposis (FAP), an inherited form of colorectal/intestinal cancer^21^. Here, we have used this mouse model to test the hypothesis that ME1 overexpression would lead to increased tumor burden. We characterized the male progeny of the novel intercross of heterozygous male Apc^Min/+^ mice with female ME1-Tg mice, namely, Apc^Min/+^ mice with intestine-specific augmentation of ME1 (designated Apc^Min/+^/ME1-Tg) for tumor parameters and for expression of candidate tumor-associated genes. Further, we utilized small molecule inhibitors of ME1 and the canonical Wnt signaling pathway, respectively to elucidate single and combinatorial effects on two human CRC cell lines. Results document a stimulatory role for ME1 in intestinal tumor genesis.

## Results

### Effects of enhanced intestinal epithelial ME1 expression

**Figure 1:**
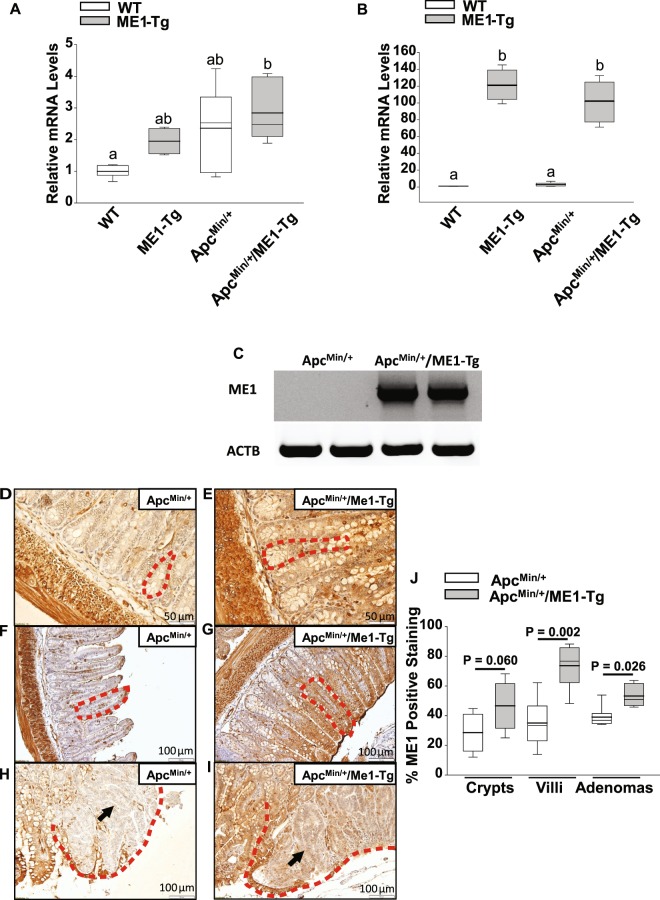
ME1 expression in Apc^Min/+^/ME1-Tg mouse intestines. (**A**) qRT-PCR of mouse *Me1* RNA in the jejunums of WT, ME1-Tg, Apc^Min/+^ and Apc^Min/+^/ME1-Tg male mice (n = 4–6/group). (**B**) qRT-PCR of total *Me1* (endogenous + transgene-derived) mRNA in the jejunums of WT, ME1-Tg, Apc^Min/+^ and Apc^Min/+^/ME1-Tg male mice (n = 4–6/group). One-Way ANOVA was used to examine for differences between groups (All Pairwise Multiple Comparison Procedures; Holm-Sidak method). Different lowercase letters (a, b) designate groups that differ (P < 0.05); however, bars sharing the same letter are not different. (**C**) Conventional RT-PCR of transgene-expressed *Me1* mRNA in Apc^Min/+^ and Apc^Min/+^/ME1-Tg male jejunums (n = 2/group). Gel lanes were cropped for purposes of space. The corresponding full-length gels are presented in Supplementary Fig. 7. (**D** to **I**) Representative ME1 IHC of Swiss-rolled ilea from Apc^Min/+^ and Apc^Min/+^/ME1-Tg male mice. (**D,E**) crypts (scale bar = 50 μm); (**F, G**) villi (scale bar = 100 μm); (**H,I**) adenomas (scale bar = 100 μm). Red dotted lines delineate crypts (**D,E**), villi (**F,G**) and adenomas (**H,I**). Black arrows point to centers of adenomas (**H,I**). (**J**) Percentage ME1-positive staining of crypts, villi (epithelium and *lamina propria*) and adenomas in ilea of male mice. Quantification was via Aperio Imagescope. Boxes demarcate the inter-quartile range of 25–75% with mean (thick line) and median (thin line); (n = 6/group). (**J**) Student’s *t*-tests were used to examine for differences in IHC staining intensity of ME1 protein between groups, and the Mann-Whitney Rank Sum Test was used for comparing non-normally distributed data. Significant differences were identified by P < 0.05. A tendency for a difference also is indicated (0.1 > P > 0.05).

To generate Apc^Min/+^ mice with enhanced intestinal expression of ME1, heterozygous male Apc^Min/+^ mice were intercrossed with female ME1-Tg mice. Sixteen-week-old male mouse progeny were used to quantify *Me1* RNA abundance and adenoma burden in the small and large intestines. We observed a significant (~2.0-fold; P = 0.010) increase in expression of the endogenous (mouse) *Me1* gene in the jejunums of Apc^Min/+^/ME1-Tg mice when compared to WT mice (Fig. 1A). Similarly, total *Me1* mRNA levels (i.e., endogenous plus transgenic *Me1* RNAs) in mouse jejunum were significantly greater for ME1-Tg (P < 0.001) and Apc^Min/+^/ME1-Tg (P < 0.001) mice when compared to WT and Apc^Min/+^ mice, respectively (Fig. 1B). As expected, no transgene-derived RNA was observed in the non-transgenic Apc^Min/+^ mouse intestine (Fig. 1C). We then evaluated relative abundance of ME1 protein in ileum by immunohistochemistry (IHC). An increase in ME1 protein (IHC staining) was observed within normal-appearing villi of the transitional mucosa (P = 0.002) as well as in adenomas (P = 0.026) of Apc^Min/+^/ME1-Tg when compared to Apc^Min/+^ mice; by contrast, the crypts of both mouse lines did not differ (Fig. 1D–J). The intestine smooth muscle layers (outer longitudinal and inner circular) stained intensely for ME1 (Fig. 1D–G); although, as expected, this staining was unaffected by *Me1* transgene. Apc^Min/+^/ME1-Tg mice exhibited greater amounts of ME1 protein in adenomas when compared to those of Apc^Min/+^. Interestingly, the borders of adenomas exhibited significantly greater ME1 staining than the corresponding inner regions irrespective of genotype (Supplementary Fig. 1). However, the adenoma borders of Apc^Min/+^/ME1-Tg mice displayed significantly greater (P = 0.042) ME1 staining than those of Apc^Min/+^ mice (Supplementary Fig. 1).

Goblet cells are the most abundant secretory cell type in the villus epithelium, and their numbers serve as a readout of lineage determination in intestines. We performed Alcian blue histochemistry to evaluate goblet cell numbers as a function of ME1 status. There was no difference in number of goblet cells in villi of Apc^Min/+^/ME1-Tg and Apc^Min/+^ mice (Supplementary Fig. 2). Villi immediately adjacent to adenomas had significantly more goblet cells (P < 0.01) than those that were more distant from adenomas (Supplementary Fig. 2). However, the number of goblet cells in adenoma-associated villi of Apc^Min/+^/ME1-Tg *vs*. Apc^Min/+^ mice did not differ (Supplementary Fig. 2).

### Increased intestinal adenoma burden in male Apc^Min/+^/ME1-Tg mice

**Figure 2:**
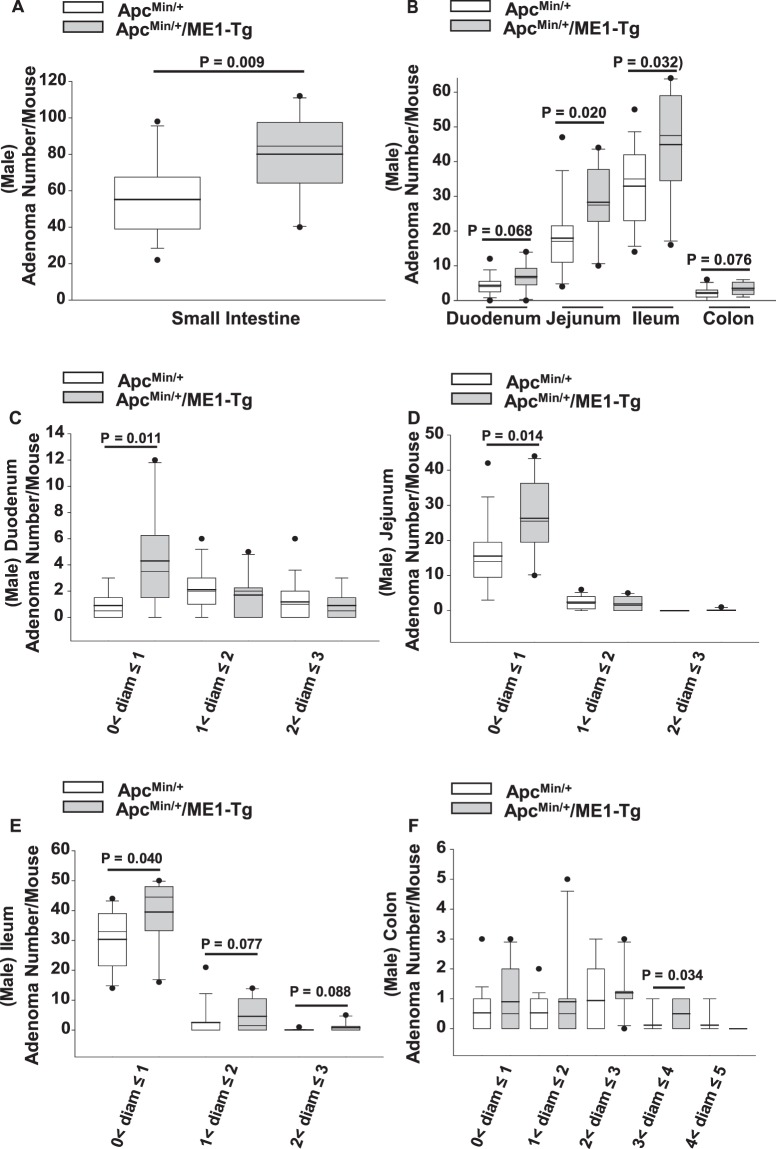
Adenoma burden in male Apc^Min/+^/ME1-Tg mice. (**A**) Total number of adenomas/mouse in the small intestines of sixteen-week-old Apc^Min/+^ (n = 17) and Apc^Min/+^/ME1-Tg (n = 10) male mice. (**B**) Regional distribution of adenomas in male mice. (**C–F**) Size distribution of adenomas in the (**C**) duodenum, (**D**) jejunum, (**E**) ileum and (**F**) colon of sixteen-week-old male Apc^Min/+^ and Apc^Min/+^/ME1-Tg mice. Boxes indicate the inter-quartile range (25–75%) with mean (thick line) and median (thin line); whiskers: 10^th^ and 90^th^ percentiles; dots: outliers. Student’s *t*-tests were used to examine for differences between groups and the Mann-Whitney Rank Sum Test was used for comparing non-normally distributed data. Significant differences were identified by P < 0.05. Tendencies for differences also are indicated (0.1 > P > 0.05).

We next evaluated the effect of the Me1 transgene on intestinal adenoma burden. Male Apc^Min/+^/ME1-Tg mice exhibited a significant increase (~1.5-fold; P = 0.009) in numbers of small intestine adenomas when compared to Apc^Min/+^ mice (Fig. 2A); this increase occurred in the Ileum (P = 0.032) and jejunum (P = 0.020) (Fig. 2B). The number of colon adenomas did not differ (P = 0.076) between Apc^Min/+^/ME1-Tg and Apc^Min/+^ mice (Fig. 2B). Apc^Min/+^/ME1-Tg mice displayed significantly greater numbers of adenomas that were less than 1 mm in diameter in the duodenum (P = 0.011), jejunum (P = 0.014), and ileum (P = 0.040) when compared to male Apc^Min/+^ mice (Fig. 2C–E). Interestingly, the number of adenomas between 3 mm and 4 mm in diameter was significantly greater (P = 0.034) in the colons of male Apc^Min/+^/ME1-Tg mice compared to Apc^Min/+^ mice (Fig. 2F).

### β-catenin IHC of adenomas

**Figure 3:**
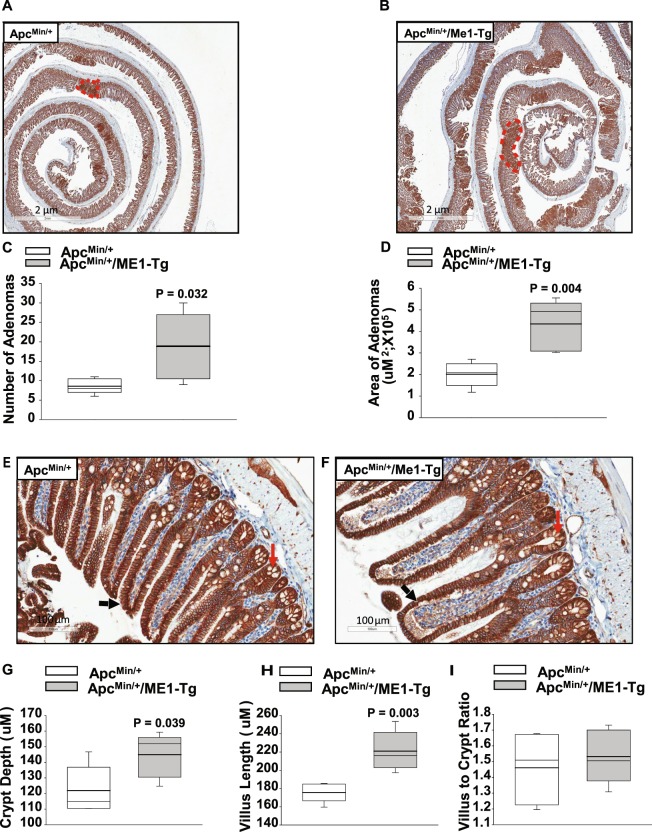
Increased numbers and sizes of ileal adenomas in sixteen-week-old male Apc^Min/+^/ME1-Tg mice. (**A**,**B**) Representative β-catenin IHC of ilea from Apc^Min/+^ and Apc^Min/+^/ME1-Tg male mice (scale bar = 2 μm). A representative adenoma is outlined in red in each panel. (**C**) Total number of ileal adenomas quantified after β-catenin IHC of male mice (n = 5/group). (**D**) Area of ileal adenomas (total adenoma area per mouse) quantified after β-catenin IHC (n = 5 male mice/group). (**E**,**F**) Representative images of β-catenin-stained ileum showing normal appearing crypts and villi (scale bar = 100 μm). Black and red arrows indicate villi and crypts, respectively. (**G**) Quantification of ileal crypt depth in Apc^Min/+^ and Apc^Min/+^/ME1-Tg male mice (n = 5/group). (**H**) Quantification of ileal villus length in male mice (n = 5/group). (**I**) Ratio of villus length to crypt depth in the ilea of male mice (n = 5/group). Boxes indicate the inter-quartile range of 25–75% with mean (thick line) and median (thin line); whiskers extend to the 10^th^ and 90^th^ percentiles. Student’s *t*-tests were used to examine for differences between genotypes (significant difference, P < 0.05).

An increase in nuclear β-catenin content is a hallmark of intestinal tumorigenesis. We therefore evaluated, by IHC, the number of nuclear β-catenin-positive cells in ilea from male Apc^Min/+^/ME1-Tg and Apc^Min/+^ mice. No significant differences in numbers of nuclear β-catenin-positive cells were observed for crypts, villi or adenomas as a function of genotype (Supplementary Fig. 3). However, all adenomas stained very strongly for β-catenin (nuclear and cytoplasmic/membranes) (Fig. 3A,B), which facilitated the measurements of adenoma areas (in cross-section) by use of Aperio software. Both adenoma number and area were significantly greater (P = 0.032 and P = 0.004, respectively) for ilea of male Apc^Min/+^/ME1-Tg mice compared to male Apc^Min/+^ mice (Fig. 3C,D). In addition, ileal crypts and villi of male Apc^Min/+^/ME1-Tg mice were significantly deeper (P = 0.039) and longer (P = 0.003), respectively when compared to those of male Apc^Min/+^ mice; however, villus to crypt ratio was comparable between genotypes (Fig. 3E–I). We examined whether the increases in crypt depth and villus length were due to increased numbers of cells within the corresponding epithelium. The number of cells lining the crypts did not significantly differ as a function of genotype; however, we observed a significant increase (~1.2-fold; P = 0.044) in the number of cells comprising the villus epithelium in the Apc^Min/+^/ME1-Tg relative to Apc^Min/+^ mice (Supplementary Fig. 3).

### Gene expression, proliferation and apoptosis in male Apc^Min/+^/ME1-Tg mouse intestines

**Figure 4:**
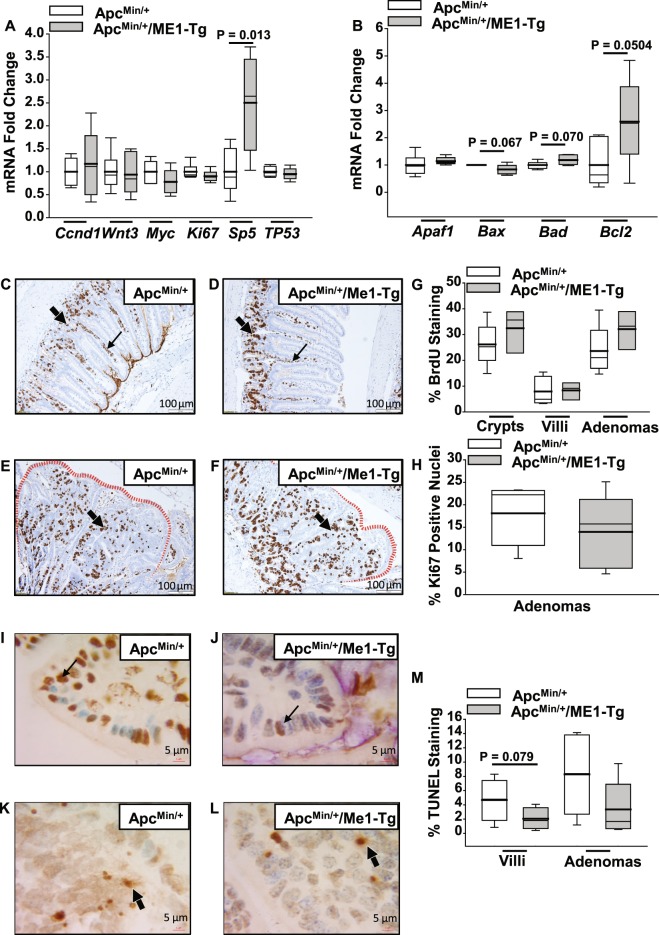
Gene expression, proliferation and apoptosis in experimental mice. (**A**) Fold change of abundance of mRNAs encoding oncogenes and tumor suppressors in jejunums of Apc^Min/+^ and Apc^Min/+^/ME1-Tg male mice (n = 5–6/group). (**B**) Fold change of mRNAs encoding apoptosis-related proteins in mouse jejunums (n = 5–6 mice/group). (**C,D**) Representative images of BrdU-labeled crypts and villi in ilea of male mice. Thin and thick arrows indicate villus and crypt, respectively. (**E,F**) Representative BrdU-labeled/stained adenomas of male mice. Red dotted lines highlight adenoma borders. Arrows indicate adenoma centers (scale bar = 100 μm). (**G**) Quantification of percentage BrdU-positive cells in ileal crypts, villi, and adenomas of male mice (n = 3–5 mice/group). (**H**) % of cells with nuclear *Ki*67 IHC staining in ileal adenomas of male mice (n = 5 mice/group). (**I,J**) TUNEL-positive cells in villi of male Apc^Min/+^ and Apc^Min/+^/ME1-Tg mice, respectively (scale bar = 5 μm). Arrows identify representative TUNEL-positive cells. (**K,L**) TUNEL-positive cells in ileal adenomas of male Apc^Min/+^ and Apc^Min/+^/ME1-Tg mice (scale bar = 5 μm). Arrows indicate representative TUNEL-positive cells. (**M**) Quantification of percentage-positive ileal TUNEL staining (n = 5–6 mice/group). Boxes indicate the inter-quartile range of 25–75% with mean (thick line) and median (thin line); whiskers extend to 10^th^ and 90^th^ percentiles. Student’s *t*-tests were used to examine for differences between groups and the Mann-Whitney Rank Sum Test was used for non-normally distributed data. Significant differences were identified by P < 0.05. Tendencies for differences also are indicated (0.1 > P > 0.05).

We performed targeted gene expression analysis to identify a pathway-oriented basis for the increased intestinal adenoma burden of Apc^Min/+^/ME1-Tg mice. Among the oncogenes and tumor suppressor genes that were examined, only *Sp5* was significantly (P = 0.013) altered (i.e., upregulated by more than two-fold) in the Apc^Min/+^/ME1-Tg jejunum (Fig. 4A). *Sp5* is a known induced target gene of the Wnt pathway^22–24^. Expression analysis of apoptosis-associated genes showed a two-fold up-regulation (P = 0.05) of anti-apoptotic Bcl2 in Apc^Min/+^/ME1-Tg mice (Fig. 4B). No differences in expression of several epithelial to mesenchymal (EMT)-associated genes were noted between the two groups (Supplementary Fig. 4). The jejunal expression of other Sp/Klf family member genes that were previously implicated in intestinal growth and homeostasis, did not differ between the mouse lines (Supplementary Fig. 4). Moreover, the numbers of BrdU-positive and nuclear Ki67-positive cells were comparable between genotypes for the crypt and villus epithelium and adenomas (Fig. 4C–H). Evaluation of apoptosis by TUNEL revealed tendencies for decreased staining in the villi and adenomas of Apc^Min/+^/ME1-Tg compared to Apc^Min/+^ mice (Fig. 4I–M).

### Increased KLF9 protein abundance in crypts and villus *lamina propria* of male Apc^Min/+^/ME1-Tg mice

**Figure 5:**
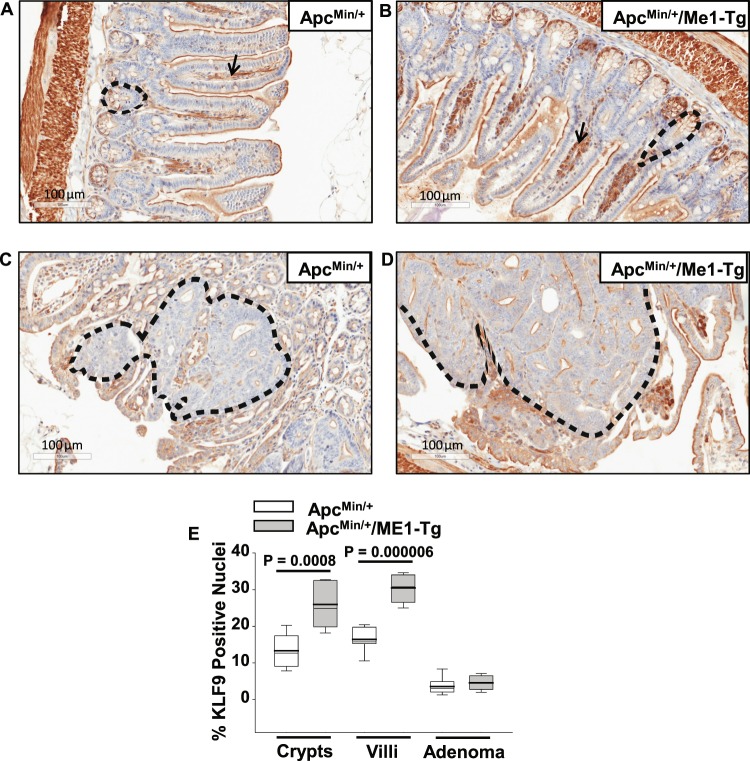
Increased KLF9 immunoreactivity in ileal crypts and lamina propria of sixteen-week-old male Apc^Min/+^/ME1-Tg mice. (**A,B**) Representative images of KLF9-positive cells in crypts (black dotted line), villi (epithelium and *lamina propria*), and *muscularis* of Apc^Min/+^ and Apc^Min/+^/ME1-Tg male mice (scale bar = 100 μm). Black arrows indicate representative KLF9 staining in the *lamina propria*. (**C,D**) KLF9 IHC of ileal adenomas from male mice (scale bar = 100 μm). Representative adenomas are outlined. (**E**) Quantification of KLF9 IHC results (n = 7 mice/group). Quantification was via the Aperio Imagescope nuclear algorithm. Boxes demarcate the inter-quartile range of 25–75% with mean (thick line) and median (thin line); whiskers: 10^th^ and 90^th^ percentiles. Student’s *t*-tests were used to examine for differences between genotypes.

We previously reported that null mutation of the transcription factor Krϋppel-like factor 9 (Klf9) negatively affected small intestine crypt stem-progenitor cell proliferation and villus cell migration in mice^25^. As a consequence, the villi of Klf9 knockout mice were shorter than their wild-type counterparts^25^. In view of the positive effect of ME1 transgene on villus length, we evaluated, by IHC, the presence of nuclear-localized KLF9 in the ilea of Apc^Min/+^/ME1-Tg and Apc^Min/+^ mice. Nuclear KLF9 protein levels were significantly greater in the crypts (P = 0.0008) and the villus lamina propria (P = 0.000006) of Apc^Min/+^/ME1-Tg compared to Apc^Min/+^ mice (Fig. 5A–E). Nuclear KLF9 immunoreactivity was minimal in the villus epithelium, but was robust in the *muscularis externa* of both genotypes (Fig. 5A,B). Moreover, KLF9 immunostaining in adenomas was negligible in both mouse lines (Fig. 5C–E).

### Inhibition of ME1 activity suppresses growth of human CRC cells *in vitro*

**Figure 6:**
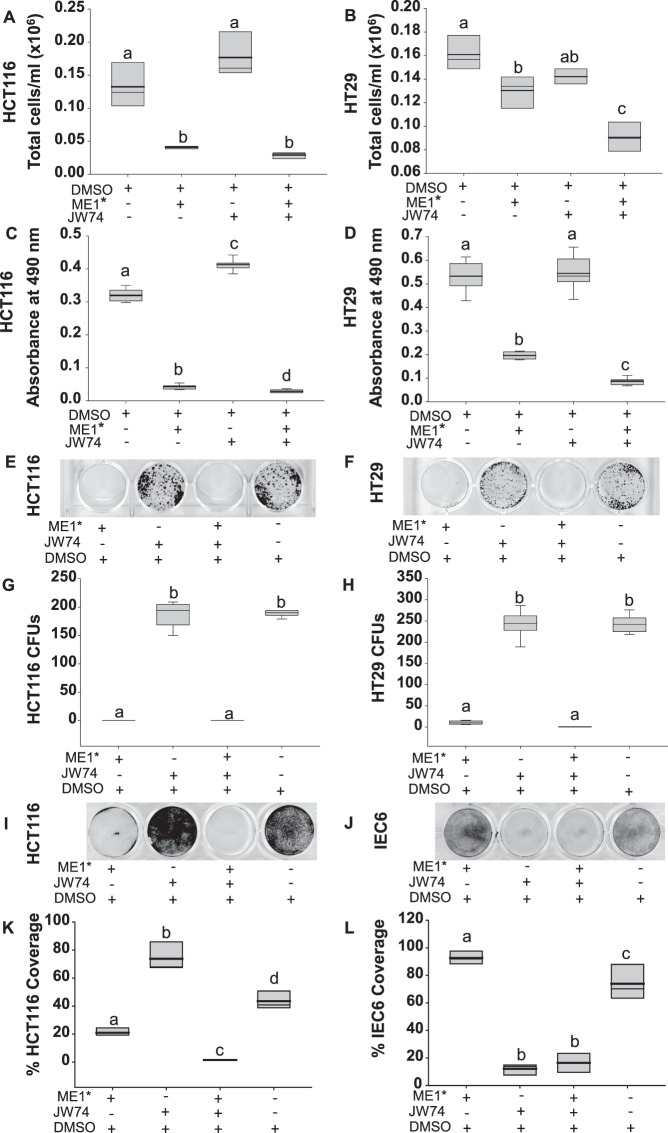
A small molecule inhibitor of ME1^26^ suppressed colon cancer cell numbers *in vitro*. (**A,B**) Number of viable HCT116 and HT29 cells after treatment with 50 uM of ME1 inhibitor (ME1*), 15 uM of Wnt pathway inhibitor (JW74), 50 uM ME1* plus 15 uM JW74, or vehicle (DMSO). Twenty thousand cells/well were plated and 24 h later received inhibitor(s). Cells were evaluated after 72 h of treatment (n = 3 wells/treatment group, data are representative of 2 independent experiments). (**C,D**) Results of MTS cell proliferation/cytotoxicity assay. Cells were plated at a density of 1000 cells per/well and 24 h later received treatments (50 uM ME1*, 15 uM JW74, 50 uM ME1* plus 15 uM JW74, or vehicle (DMSO)). Absorbance (490 nm) was measured at 48 h after treatment addition; n = 8 wells/treatment group, data are representative of 2 independent experiments. (**E,F**) Results of clonogenic assay. HCT116 or HT29 cells were plated at a density of 1000 cells/well and after 24 h were treated with 50 uM ME1*, 15 uM JW74, 50 uM ME1* plus 15 uM JW74, or vehicle (DMSO). After incubation for six days, cells were stained with crystal violet. (**G,H**) Quantification of colony forming units (CFU) from (**E**,**F)** after treatment of cells (n = 6 wells/treatment group). (**I,J**) Results of modified clonogenic assay. HCT116 and IEC6 cells were plated at very high density (100,000 cells/well) and after 24 h were treated with 50 uM ME1*, 15 uM JW74, 50 uM ME1* plus 15 uM JW74, or vehicle (DMSO). After 3 days, cells were stained with crystal violet. (**K,L**) Quantification of remaining cells from (**I**,**J**) expressed as % area of stained cells per well. Boxes show the inter-quartile range of 25–75% with mean (thick line) and median (thin line); whiskers: 10^th^ and 90^th^ percentiles. One way ANOVA was used to examine for differences between treatment groups. Different lowercase letters (a-d) designate groups that differ (P < 0.05); bars sharing the same letter are not significantly different.

We next evaluated the involvement of ME1 in the Wnt/β-catenin pathway using intestinal cell lines. In initial studies, we inhibited ME1 enzyme activity in the non-cancerous IEC6 intestinal epithelial cell line with a small molecule inhibitor of ME1^26^ (designated ME1*). A small but significant decrease in colony formation was noted for these cells but only at the highest dose evaluated (Supplementary Fig. 5). Since our earlier results for *Sp5* (Fig. 4A) indicated a possible functional connection between ME1 and the canonical Wnt/β-catenin signaling pathway, we next treated HCT116 and HT29 CRC cells, singly and in combination, with the small molecule ME1 inhibitor and an inhibitor of the canonical Wnt pathway (JW74)^27^. Non-confluent cells were treated with 50 uM ME1*, 15 uM JW74, 50 uM ME1* plus 15 uM JW74, or vehicle (DMSO; control) for 72 h. Results showed a significant reduction in total cell numbers with ME1* or ME1* + JW74 for both HCT116 and HT29 cell lines (Fig. 6A,B). In HT29 cells, the combination of ME1* and JW74 had an additive inhibitory effect on cell counts compared to vehicle (P < 0.001) (Fig. 6B). JW74 alone did not alter cell numbers (Fig. 6A,B). Diameters of HCT116 and HT29 cells treated with ME1* or ME1* + JW74 were reduced when compared to vehicle; JW74 alone did not affect HCT116 cell diameter but showed a tendency for this in HT29 cells (P = 0.067) (Supplementary Fig. 5). We also examined HCT116 and HT29 cell viability/metabolism by use of the MTS reagent. A significant reduction in cell viability/metabolic activity was observed with ME1* treatment, which was further diminished with co-addition of JW74 (Fig. 6C,D). Unexpectedly, JW74 alone had a small but significant (P < 0.001) stimulatory effect on HCT116 cells.

We performed colony formation assays to confirm the above effects. ME1* and ME1* + JW74 dramatically reduced (P < 0.001) the number of colonies formed by both cell lines, while JW74 alone had no effect (Fig. 6E–H). ME1* dose-dependently reduced the number of colonies formed by both HCT116 (overall P < 0.001) and HT29 (overall P < 0.001) cells (Supplementary Fig. 5), albeit HCT116 cells were more sensitive than HT29 cells to ME1*. Since the number of colonies formed after treatment with ME1* or ME1* + JW74 were so few, we inferred that the ME1 inhibitor was causing cell death. To investigate this further, HCT116 cells were seeded at high density and treated with ME1*, JW74, ME1* + JW74, or vehicle (DMSO). ME1* caused significant loss of cells, while the combination of ME1* and JW74 showed an additive effect on cell numbers (Fig. 6I,K). IEC6 cells when treated similarly showed no effect with ME1* while JW74 alone and combination of JW74 and ME1* had comparable inhibitory effects (Fig. 6J,L).

### ME1 expression in human colon adenocarcinomas

ME1 was immunolocalized to specific cells of human normal colon and colon tumor tissue using a commercial tissue array. Robust expression of ME1 was observed in the more differentiated epithelial regions of both human colorectal adenocarcinomas and normal colon (Supplementary Fig. 6).

## Discussion

This study reports a novel *in vivo* association between gastrointestinal ME1 expression and small intestine adenoma burden. We observed increases in: a) adenoma number and size distribution, b) ME1 abundance within adenomas, and c) crypt depth and villus height, in the non-adenoma (transitional) mucosa with *Me1* overexpression in the background of *Apc* haplo-insufficiency. We also found a significant increase in *Sp5* transcript levels and enhanced numbers of nuclear KLF9 positive-cells in the crypt epithelium and *lamina propria* of Apc^Min/+^/ME1-Tg mice. These findings link an important cytosol-residing metabolic enzyme namely ME1 with alterations in expression (and by inference, downstream pathway effects) of two members of the SP/KLF family of transciptional regulators, which are themselves increasingly considered as context-dependent participants in oncogenesis and canonical Wnt pathway signaling. Moreover, our *in vitro* experiments utilizing two human CRC cell lines confirmed that ME1 is important for cancer cell growth (hyperplasia and hypertrophy). Importantly, we found that growth of cancer cells was highly sensitive to inhibition of ME1 enzyme activity; by contrast, growth of non-tumorigenic intestinal epithelial cells (IEC6) was relatively resistant to ME1 inhibition albeit sensitive to Wnt pathway inhibition. Finally, we showed that ME1 is abundantly expressed within epithelial-like regions of human colon adenocarcinoma akin to what was observed in the adenoma borders in Apc^Min/+^/ME1-Tg mice. Collective results implicate ME1 as a functional contributor to intestinal cancer development and suggest that targeting ME1 should be explored as a potential therapeutic strategy to improve patient outcome.

Analogous to our previous findings for ME1-Tg mice^19^, the Apc^Min/+^/ME1-Tg mice exhibited greater ME1 RNA and protein levels in the small intestine, when compared to their littermate controls. The greater numbers of small adenomas and the larger adenoma sizes, respectively within the small intestine of Apc^Min/+^/ME1-Tg mice, coincident with increased ME1 expression, are suggestive of ME1 promotion of the initial step(s) of adenoma formation and of ME1 participation in tumor progression. Moreover, the significant elevation in ME1 abundance within the adenoma borders is consistent with previously reported increases in lipid content in the epithelial-like adenoma borders of Apc^Min/+^ mice^28^.

The lack of differences in the expression of proliferation-associated genes, which were corroborated by results of BrdU and *Ki*67 staining, between the two mouse lines with distinct intestinal ME1 expression, suggest that the increased adenoma burden *in vivo* may not result from increased cell proliferation but rather to decreased apoptotic status. Consistent with this, we found an increase in anti-apoptotic *Bcl2* gene expression in the jejunum and a decrease in TUNEL staining in the villi of Apc^Min/+^/ME1-Tg mice. The lack of observed changes in the expression of several canonical EMT-associated genes may be related to EMT occuring at later stages of tumorigenesis than studied here or to the lack of functional relationship of ME1 and EMT. Further studies conducted at later stages of tumorigenesis should address this question.

Both crypt depth and villus length were enhanced in the transitional mucosa of Apc^Min/+^/ME1-Tg mice. We found no increase in the number of cells resident in the crypt epithelium of Apc^Min/+^/ME1-Tg mice, although the average number of cells per crypt was numerically greater than for Apc^Min/+^ mice; thus, a combination of increased cell number and cell size likely contributed to the increased crypt depth. Consistent with this, ME1 inhibition conferred smaller cell diameters *in vitro*. Moreover, in a previous study, we reported that mice functionally null for *Me1* had shallower colon crypts when fed a high fat diet^20^. By contrast, ME1-Tg mice (on the WT *Apc* background) fed a high-fat diet exhibited increased jejunum crypt depth and more crypt stem-progenitor proliferation then wild-type littermate controls^19^. The ME1-Tg mice on the WT *Apc* background and fed a high fat diet, also had altered liver metabolism, reflecting gut-liver communication^19^; similar effects may have contributed to the current findings. We conclude that ME1 may play a role, either directly or indirectly, in the maintenance of intestinal crypt stem-progenitor cell number and/or size.

We noted a virtual absence of goblet cells within the central regions of adenomas, whereas their borders contained a high frequency of these cells. While we found no quantitative effects of the *Me1* transgene on this pattern, there was a significant increase in the number of goblet cells in the adenoma-associated villi when compared to normal villi. Perhaps the greater number of goblet cells within the transitional mucosa reflects a tumor-protective function through promotion of mucus production and of paracrine signaling elicited by tumor-promoting mucins^29^.

Our studies implicate two members of the Sp/Klf-family of transcription factors as potential mediators of ME1-induced GI tumorigenesis. SP5, a downstream target of the Wnt/β-catenin signaling pathway in colon and several other tissues, is reported to be up-regulated in CRC and to promote tumor cell growth^22–24^. Given that *Sp5* transcript levels were elevated in the jejunums of Apc^Min/+^/ME1-Tg mice yet we observed no differences in nuclear β-catenin localization in the two mouse lines, we infer that the *Sp5* induction in response to ME1 occured via an alternate pathway or via interconnectivity with the Wnt/β-catenin signaling pathway but downstream of nuclear β-catenin. Alternatively or in addition, the increase in *Sp5* mRNA abundance may reflect increased numbers of adenoma cells expressing *Sp5* mRNA at high levels. However, since the effect of ME1 transgene on *Sp5* is relatively specific and is not accompanied by differential expression of genes (e.g., c-Myc, cyclin D1) known to be overexpressed in intestinal adenomas, the latter scenario is not likely. Future studies should address this non-canonical link between SP5 and Wnt-β-catenin signaling.

KLF9 is another potential player in ME1-enhanced tumorigenesis. In a previous study, we reported that mice null for *Klf9* had shorter intestinal villi than wild-type mice, due in part to reduced crypt cell proliferation and slower epithelial cell migration to the villus tip^25^. Thus, the increased villus length observed in the present study may be a result, in part, of the enhanced number of KLF9-positive cells in the crypts of Apc^Min/+^/ME1-Tg mice. Interestingly, numbers of nuclear KLF9-positive cells were also elevated within the villus *lamina propria* of Apc^Min/+^/ME1-Tg mice. At present, the identities of KLF9-positive cells in the villus *lamina propria* remain unknown, although this tissue compartment harbors lymphoid cells, macrophages and myofibroblasts, among other cell types. KLF9 is reported to be oncogenic in some contexts and to be tumor suppressive in others^30–33^. In a previous study, we found that *Klf9* KO caused a significant reduction in adenoma number in the colon but had no effect on adenoma number in small intestines of Apc^Min/+^ mice^33^. Taken together with the findings reported here, our results suggest the tissue and context-dependent functions of KLF9. We speculate that the enhanced frequency of KLF9-positive cells in the small intestine crypts is growth-promoting for normal appearing and transitional villi, whereas KLF9 exerts tumor-suppressive actions in adenomas of the colon but not small intestine.

Remarkably, normal rat intestinal cells (IEC6) were more resistant than cancer cells to the ME1 inhibitor but were highly sensitive to Wnt pathway inhibition; the latter is in keeping with the well-known stimulatory role of the Wnt/β-catenin signaling pathway in intestinal stem-progenitor cell proliferation. The suppressive effect of ME1 inhibitor on cancer cell size *in vitro* is consistent with our previous work with the global *Me1* hypomorphic null mouse in which we observed significant reductions in colon *Mtor* expression^20^. The mTOR pathway is dominant in determining cell size for many tissues/cells^34^; hence, reductions in its expression may partly explain our *in vitro* data.

In conclusion, our results significantly extend previous findings from other laboratories implicating a role for ME1 in gastrointestinal cancers. The body of work implicating fatty acid synthesis in CRC initiation, progression and metastasis^12–14^, coupled with the documented role of ME1 in promoting lipogenesis in gut epithelium via the NADPH supply^9,19^, further identifies this pathway as a vulnerability to exploit for CRC treatment and therapy.

## Methods

### Animals

All procedures involving mice were approved by the Institutional Animal Care and Use Committee (IACUC) of the University of Arkansas for Medical Sciences in accordance with federal guidelines and regulations. Mice were housed under a 12 h light/12 h dark cycle and were fed a regular chow diet. Apc^Min/+^ mouse breeders (Strain Name: C57BL/6J-Apc^Min^/J; Apc^Δ850^, stock number: 002020) were from the Jackson Laboratory (Bar Harbor, ME, USA). Mice with augmented intestinal epithelial expression of ME1 (rat ME1 cDNA under the control of the murine villin promoter-enhancer; ME1-Tg; C57BL/6 J background) were described previously^19^. To generate Apc^Min/+^ mice with gut-specific enhanced expression of ME1, heterozygous male Apc^Min/+^ mice were intercrossed with female ME1-Tg mice. Age- matched male mice were used to quantify tumor burden in the small and large intestines. At 16 weeks of age, mice (n = 17 Apc^Min/+^ male mice and n = 10 Apc^Min/+^/ME1-Tg male mice) were euthanized for tissue collection. Their small intestines and colons were removed, flushed with phosphate-buffered saline (PBS) and opened longitudinally. The small intestine was divided into three equal parts, which were operationally designated as duodenum, jejunum, and ileum. The junction (~1 cm in length) between the jejunum and ileum was snap-frozen in liquid nitrogen and stored at −80 °C for later analysis. Jejunum-ileum junctions (referred to as jejunum in Results) were also obtained from age-matched non-transgenic wild-type and ME1-Tg male mice (both strains wild-type for *Apc*). After microscopic examination of tissues (below), each Ileum was coiled into a Swiss-roll and fixed in methanol Carnoy solution (60% methanol, 30% chloroform, 10% glacial acetic acid) for 24 h and then transferred to 70% ethanol, followed by embedding in paraffin. We chose ilea for embedding and subsequent IHC, since this region typically displays the most adenomas in Apc^Min/+^ mice^33^. To evaluate gastrointestinal proliferation, Apc^Min/+^/ME1-Tg and Apc^Min/+^ mice were injected intraperitoneal with BrdU at a dose of 100 mg/kg of body weight (Sigma Aldrich, St. Louis, MO, USA) 2 h before euthanizing, as described previously^20^.

### Microscopic examination of adenomas

Intestine segments and colons were examined for number and size of adenomas, in blinded fashion, with a dissecting microscope (Discovery 8, Carl Zeiss MicroImaging GmbH, Jena, Germany). All adenomas were counted, measured and categorized by size (0 < diam ≤ 1 mm, 1 < diam ≤ 2 mm, 2 < diam ≤ 3 mm, 3 < diam ≤ 4 mm, 4 < diam ≤ 5 mm (diam = diameter)).

### Quantitative Real-Time, Reverse Transcriptase-Polymerase Chain Reaction (qRT-PCR)

RNA was extracted from each jejunum using TRIzol reagent (Invitrogen, Waltham, MA, USA). Using the iScript cDNA synthesis kit (Bio-Rad; Hercules, CA, USA), one microgram of RNA was reverse-transcribed into cDNA. Gene expression was evaluated by qRT-PCR using Bio-Rad iTaq SYBR Green Supermix. Primers (Table 1) were procured from Integrated DNA Technologies, Inc. (Coralville, IA, USA). Using GeNorm software^35^, target mRNA levels were normalized to a factor calculated from the geometric mean of expression values for β-Actin (*Actb*), Cyclophilin A (*Ppia*) and TATA box binding protein (*Tbp*). For detection of the chimeric *Me1* transgene RNA in the small intestine by conventional RT-PCR, a primer pair was used to amplify a segment spanning the SV40 poly A and *Me1* coding regions [forward primer (*Me1*): 5′-AAT GAT TCG GTCTTC CTC ACC-3′, and reverse primer (SV40): 5′-CAG ACA TGA TAA GAT ACA TTG ATG AGT T-3′] of the transgene construct.

**Table 1:** DNA primers used in qRT-PCR of mouse genes.

*Mouse gene*	Forward primer (5′-3′)	Reverse primer (3′-5′)
*Actb*	ACCTTCTACAATGAGCTGCG	CTGGATGGCTACGTACATGG
*Apaf1*	GATGTGGAGGTGATCGTGAAG	TACTGGATGGTGCTGTGATG
*Bad*	AGGATGAGCGATGAGTTTGAG	CCTTTGCCCAAGTTTCGATC
*Bax*	TTGGAGATGAACTGGACAGC	CAGTTGAAGTTGCCATCAGC
*Bcl2*	GGACTTGAAGTGCCATTGGTA	GTTATCATACCCTGTTCTCCCG
*Ccnd1*	GCCCTCCGTATCTTACTTCAAG	GCGGTCCAGGTAGTTCATG
*Cdh1 (E-Cad)*	AGAGAAGCCATTGCCAAGTAC	AACGAATCCCTCAAAGACCG
*Cdh2 (N-CAD)*	CTTCCTTGCTTCTGACAATGG	TGAGTTGGGTTCTGGAGTTTC
*Ki67*	TGCCCGACCCTACAAAATG	GAGCCTGTATCACTCATCTGC
*Klf4*	ACTTGTGACTATGCAGGCTG	ACAGTGGTAAGGTTTCTCGC
*Klf5*	TCGTCTCACTTAAAAGCTCACC	CCGTGTGCTTCCTGTAGTG
*Klf9*	GGC TGT GGG AAA GTC TAT GG	AGT GTG GGT CCG GTA GTG
*Klf13*	TATGTGGACCACTTTGCCGCC	TGCTGGTTGAGGTCCGCTAGGAT
*Klf15*	CACAAATGCACTTTCCCAGG	TTGACAACTCATCTGAGCGG
*Me1*	AGTATCCATGACAAAGGGCAC	ATCCCATTACAGCCAAGGTC
*Me1 (rat)*	CAACTCCTATGTGTTCCCTGG	TGACACTTGCTGGGATATGAC
*Myc*	GCT GTT TGA AGG CTG GAT TTC	GAT GAA ATA GGG CTG TAC GGA G
*Ppia*	GCAGACAAAGTTCCAAAGACAG	CATTATGGCGTGTAAAGTCACC
*Snai1*	GTGAAGAGATACCAGTGCCAG	AAGATGCCAGCGAGGATG
*Sp5*	AGAAGCTCAAAGTCGCTGAG	AAGGTGCTGGGAAAGATGTC
*Tbp*	AAGAAAGGGAGAATCATGGACC	GAGTAAGTCCTGTGCCGTAAG
*Tp53*	GGCGCACAGAGGAAGAGAAT	GGAGAGGAGCTGGTGTTGTTG
*Vim*	TTTCTCTGCCTCTGCCAAC	TCTCATTGATCACCTGTCCATC
*Wnt3*	AGC TGC CAA GAG TGT ATT CG	CTA GAT CCT GCT TCT CAT GGG

### Histology and immunohistochemistry

Five-micron sections of ilea (Swiss rolls) were used for immunohistochemistry (IHC). Paraffin-embedded sections were dewaxed and rehydrated through a graded alcohol series as described previously^19,20^. Antigen unmasking was conducted by boiling the sections in Coplin jars in a microwave using Citra Plus (Biogenex, San Ramon, CA, USA) for a duration of 2 min high power and then for 10 min at low power setting. After cooling for 30 min at room temperature, sections were treated with 3% hydrogen peroxide, followed by incubation in blocking solution containing goat IgG (Vectastain Elite ABC kit, Vector Laboratories, Burlingame, CA, USA) for 30 min. Sections were incubated overnight with rabbit ME1 polyclonal antibody (1∶200 dilution, Proteintech Group, Chicago, IL, USA) in a humidity chamber placed at 4 °C and then incubated with secondary goat anti-rabbit antibody (Vectastain Elite ABC kit; Vector Laboratories) for 30 min. Sections were stained with 3,3′-diaminobenzidine tetra-hydrochloride (Dako, Carpinteria, CA, USA) and counterstained with hematoxylin. The human colon cancer and normal colon tissue array (BC051110; US Biomax, Rockville, MD, USA) was similarly immunostained with rabbit ME1 polyclonal antibody. Samples on the human tissue array were obtained by the commercial vendor (with informed consent under auspices of their IRB and in accordance with federal regulations) and were de-identified prior to distribution. Other antibodies used here included a rat monoclonal anti-BrdU antibody (ab6326, 1∶40 dilution, Abcam, Cambridge, MA, USA), anti-BTEB/KLF9 antibody [(aa38–87) IHC-plus (1:200 dilution, LS-B5581, LifeSpan BioSciences, Seattle, WA, USA)], and *Ki*-67 antibody (1:200 dilution, sc-7846, Santa Cruz Biotechnology, Dallas, TX, USA).

Slides were imaged with a Aperio CS2 image capture device (Leica Biosystems Nussloch GmbH, Germany), or Nikon Eclipse E400 Microscope (Nikon Instruments, Melville, NY) fitted with an Olympus Dp73 digital camera. Quantification of positive antibody staining was performed using Aperio ImageScope algorithms or manually by counting the number of individual nuclear-stained cells. All positive staining was represented as a percentage of total staining (positive + negative) within a given field; with the exception of *Ki*67 and KLF9 for which the Aperio nuclear stain algorithm was used. Five representative areas per slide/mouse, each with 3 to 8 representative crypts or villi, were used for quantification.

### Apoptosis assay

Sections were stained using the ApopTag Peroxidase *In Situ* Apoptosis Detection kit following the manufacturer’s instructions (Millipore, Burlington, MA, USA). Staining was evaluated using the Aperio ImageScope.

### Measurement of crypts and villi

Ileal sections were scanned using the ScanScope CS2 slide scanner and Aperio ImageScope software. Crypt depths were measured from the base of the crypts to the base of the villus, while villus lengths were measured from the base of the villus to its tip. The number of cells along the sides of crypts and villi were manually counted from representative images. Three to five representative areas per slide/mouse, each with 3 to 5 representative crypts or villi, were used for quantification.

### Alcian Blue staining

Alcian Blue staining was performed using a kit (Alcian Blue (pH 2.5) stain kit H-3501, Vector Laboratories, Burlingame, CA, USA). The counter-stain was nuclear fast red. Goblet cells were manually counted from representative slides.

### Cell culture

The HCT116 and HT29 human colon cancer cell lines and the rat intestinal epithelial cell line (IEC6) were obtained from the American Type Culture Collection (ATCC, Rockville, MD, USA). Cells were maintained in Dulbecco’s Modified Eagle’s Medium (DMEM) containing 10% heat-inactivated fetal bovine serum (FBS) (Invitrogen) at 37 °C in a humidified incubator (atmosphere of 95% air/5% CO_2_). Cells were passaged using trypsin.

### Cell growth/metabolism assay

Cell proliferation/metabolism was monitored using the CellTiter 96® AQueous Non-Radioactive Cell Proliferation Assay (MTS) (Promega Corporation, Madison, WI, USA). Cells were plated at a density of 1 × 10^3^ cells/well in 96-well plates in 100 µl DMEM containing 10% heat-inactivated FBS and incubated for 24 h, followed by removal of medium, then addition of treatment [ME1 inhibitor: 3-[4-(4-hydroxyphenyl)piperazin-1-yl]-1-phenylpyrrolidine-2,5-dione^26^ (ME1*)(Vitas-M Laboratory, Ltd, Champaign, IL, USA), canonical Wnt signaling pathway inhibitor: 4-[4-(4-Methoxyphenyl)-5-[[[3-(4-methylphenyl)-1,2,4-oxadiazol-5-yl]methyl]thio]-4*H*-1,2,4-triazol-3-yl]-pyridine^27^ (JW74) (MedChemExpress; Monmouth Junction, NJ, USA), combination of ME1* and JW74, or 0.5% DMSO control, in 100 µl DMEM also containing 2% heat-inactivated FBS, followed by incubation for 48 h. All treatments contained 0.5% DMSO. Cells were then incubated with MTS/PMS solution for 4 h and absorbance was measured at 490 nm in a plate reader. All measurements were blanked against wells with the respective treatment in DMEM containing 2% FBS and 0.5% DMSO but without any cells.

### Analysis of cell numbers and cell sizes

Cells were plated in 6-well tissue culture plates at a density of 2 × 10^4^ cells in 2 ml of medium (DMEM + 10% heat-inactivated FBS) per well. After 24 h, medium was removed, and ME1 inhibitor (ME1*), canonical Wnt signaling pathway inhibitor (JW74), the combination of ME1* and JW74, or (0.5%) DMSO was added to plated cells in 2 ml of medium (containing 2% heat-inactivated FBS) and incubation continued for an additional 72 h. All treatments contained 0.5% DMSO. Wells were gently washed with PBS and then incubated with 1 ml of trypsin (Gibco™ Trypsin-EDTA (0.25%), with Phenol Red) and the entire 1 ml sample analyzed in a Vi-CELL™ XR viability counter (Beckman Coulter, Brea, CA, USA). Cell diameter was simultaneously recorded using this same instrument.

### Colony-formation assays

HT29 and HCT116 cells were plated at a density of 1 × 10^3^ cells in 1 ml/well (however, 2 × 10^3^ IEC6 cells were plated) in 24-well tissue culture plates (medium was DMEM + 10% heat-inactivated FBS) followed by incubation for 24 h. One ml containing the treatment (ME1*, JW74, the combination of ME1* and JW74, or 0.5% DMSO) were added in DMEM containing 10% heat-inactivated FBS (complete media), followed by incubation for six days (all treatment media contained 0.5% DMSO). Cells were washed with Dulbecco’s phosphate-buffered saline and stained with 0.1% crystal violet in 10% formalin for 1 h. Plates were scanned using an Epson Perfection V600 Photo Scanner at 600 DPI, and colonies counted using OpenCFU^36^ colony counting software. For OpenCFU, the threshold was set to ‘regular’ with a value of 3; minimum radius was set to 1, and maximum radius was set to auto. For IEC6 cells, colony areas were calculated using FIJI software^37^. For additional evaluation of the acute cytotoxic effects of inhibitors, cells were plated at high density (100,000 cells) per well in 12-well tissue culture plates (medium was DMEM containing 10% heat-inactivated FBS). After 24 h, treatments (ME1*, JW74, the combination of ME1* and JW74, or 0.5% DMSO) were added in 1 ml of DMEM containing 2% heat-inactivated FBS, and incubation was continued for 3 days (all treatment samples contained 0.5% DMSO). Cells were washed with Dulbecco’s phosphate-buffered saline and stained for 1 h with 0.1% crystal violet in 10% formalin. The plates were air-dried and scanned using an Epson Perfection V600 Photo Scanner at 600 DPI. Area of remaining cell coverage in each well was quantified using FIJI software as a percentage of total area of well.

### Statistics

Power analysis indicated an ability to detect a difference of 10 adenomas per mouse (per experimental group) with n = 12 animals per genotype (SD = 8, P < 0.05, power = 0.8173, two sample t-test test). To detect a 50% difference in mRNA abundance as a function of ME1 transgene at the 0.05 level required a minimum of 6 animals per group (S.D. = 0.282; power = 0.8035; two sample t-test). Statistical analysis was performed using SigmaPlot V13.0 (Systat Software, San Jose, CA, USA). One-Way ANOVA and Student’s *t*-tests were used to examine for differences between groups. The Normality Test (Shapiro-Wilk) was used to check if data were normally distributed before conducting Student’s *t*-tests. The Mann-Whitney Rank Sum Test was used to compare data between two groups that were not normally distributed. Only two-tailed P values were used and are listed in the figures. Significant differences were identified by P < 0.05.

## Electronic supplementary material

Supplementary Figures

## Data Availability

Additional data relating to the manuscript will be made available upon request.
