# Supplementary material for: Malic Enzyme 1 (ME1) is pro-oncogenic in ApcMin/+ mice
Source: Sci Rep. 2018 Sep 24;8:14268. doi: 10.1038/s41598-018-32532-w (PMC6155149; doi:10.1038/s41598-018-32532-w)
Supplement: Supplementary file 1 — Supplementary Figures [file 41598_2018_32532_MOESM1_ESM.pdf]

Malic Enzyme 1 (ME1) is pro-oncogenic in $Apc^{Min/+}$ mice

Lorenzo M. Fernandes[1,2], Ahmed Al-Dwairi[2], Rosalia C.M Simmen[1,2,3] , Meera Marji[2], Dustin M. Brown[2], Sarah W. Jewell[3] & Frank A. Simmen[1,2,3]*

Supplemental Information

Supplementary Figure 1

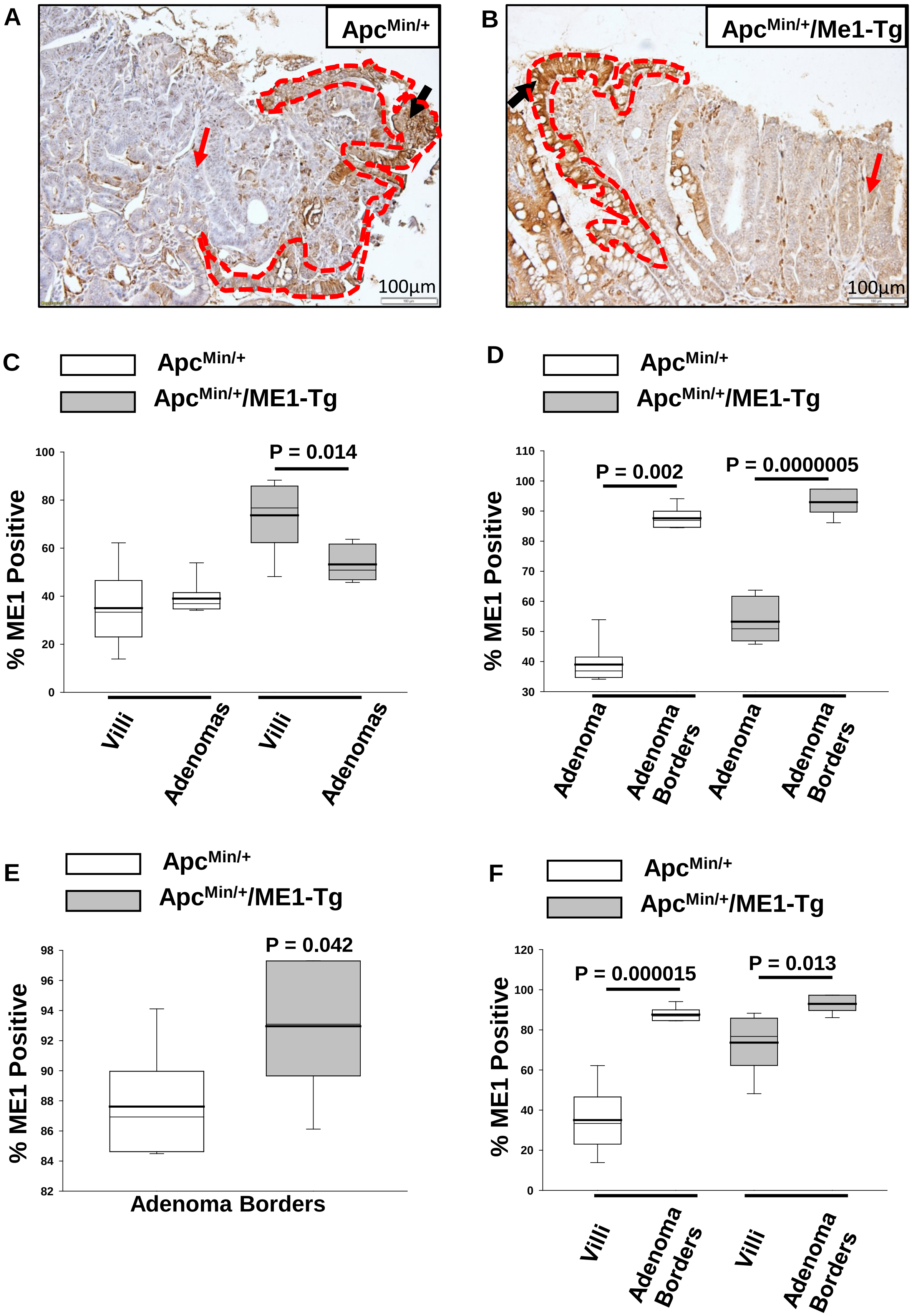

Supplementary Figure 1. Adenoma borders display relatively high levels of ME1. (**A, B**) ME1 IHC of ileal adenomas (borders outlined in red) from male mice (scale bar=100 μm). Red arrows point to centers of adenomas, whereas black arrows indicate intensely stained regions at the borders. (**C**) Comparison of percentage ME1-positivity between villi (epithelium plus *lamina propria*) and adenomas (excluding adenoma borders) of male mice. (**D**) Comparison of percentage ME1-positivity in adenomas *vs.* adenoma borders of male mice. (**E**) Comparison of percentage ME1-positivity in adenoma borders of male mice. (**F**) Comparison of percentage ME1-positivity between villi (epithelium including *lamina propria*) and adenoma borders of mice. Quantification was via Aperio Imagescope. Data are presented as boxes (inter-quartile range of 25-75%) with mean (thick line) and median (thin line); whiskers: 10th and 90th percentiles (n=6 mice/group). Student's *t*-tests were used to examine for differences in ME1 IHC staining and the Mann-Whitney Rank Sum Test was used for comparing non-normally distributed data.

Supplementary Figure 2

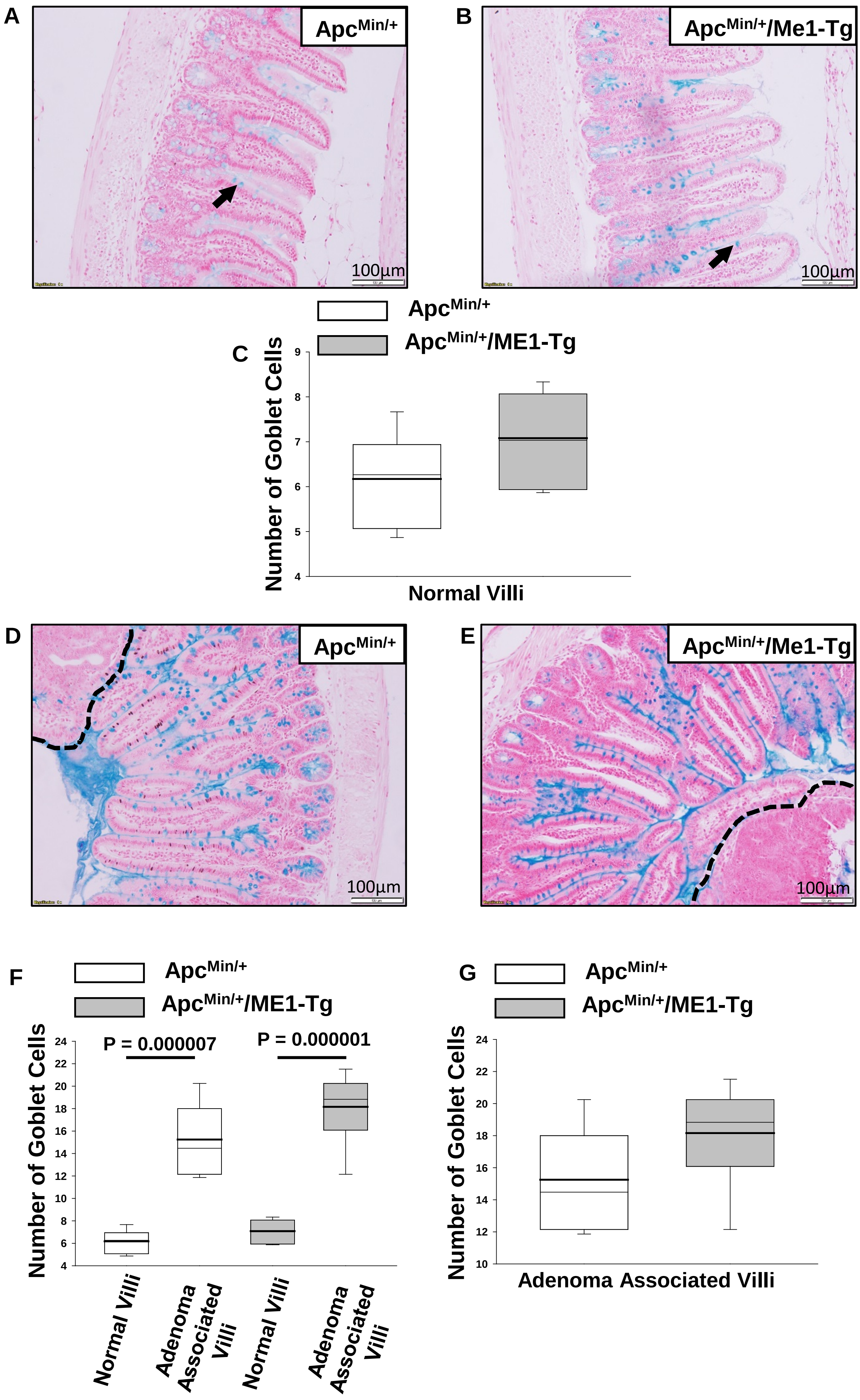

Supplementary Figure 2. Adenoma-associated villi have a higher density of goblet cells. (**A, B**) Alcian blue-stained villi of $Apc^{Min/+}$ and $Apc^{Min/+}$/ME1-Tg male mice (scale bar=100 μm). Black arrows indicate representative goblet cells. (**C**) Number of goblet cells/villus within normal appearing villi of male mice. (**D, E**) Alcian blue-stained ileal adenoma-associated villi (scale bar=100 μm). Black dotted lines delineate borders of associated adenomas. (**F**) Number of goblet cells/villus in normal villi and adenoma-associated villi of male mice. (**G**) Number of goblet cells/villus within the adenoma-associated villi of male mice. Goblet cells were counted in blinded fashion. Boxes show the inter-quartile range (25-75%) with mean (thick line) and median (thin line); whiskers: 10th and 90th percentiles (n=7 mice/group). Student's t-tests were used to examine for differences between groups. Significant differences at $P<0.05$.

Supplementary Figure 3

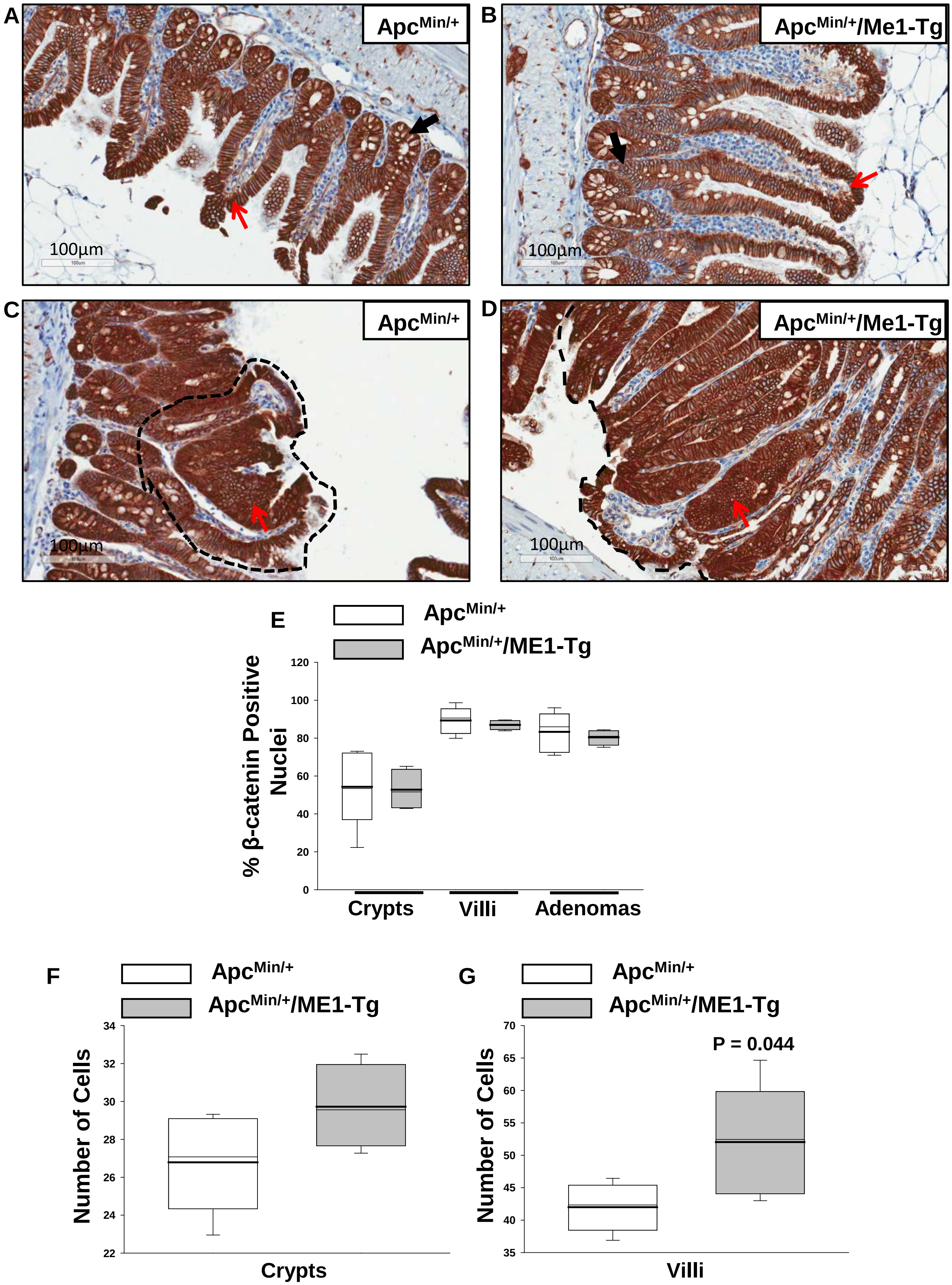

Supplementary Figure 3. IHC for β-catenin in crypts and villi (**A, B**) and adenomas (**C, D**) of $Apc^{Min/+}$ and $Apc^{Min/+}$/ME1-Tg male mice (scale bar=100 μm). Red arrows indicate villi and black arrows indicate crypts (**A, B**). Red arrows indicate centers of adenomas (**C, D**). (**E**) Quantification of nuclear β-catenin positive cells in crypt villi and adenomas. Number of cells comprising one side of crypts (**F**) and villi (**G**) of Swiss-rolled ilea from $Apc^{Min/+}$ and $Apc^{Min/+}$/ME1-Tg male mice. Boxes indicate the inter-quartile range of 25-75% with mean (thick line) and median (thin line); whiskers: 10th and 90th percentiles; n=4-5 mice/group. Student's t-tests were used to compare differences between groups. Significant differences were identified by $P<0.05$.

Supplementary Figure 4

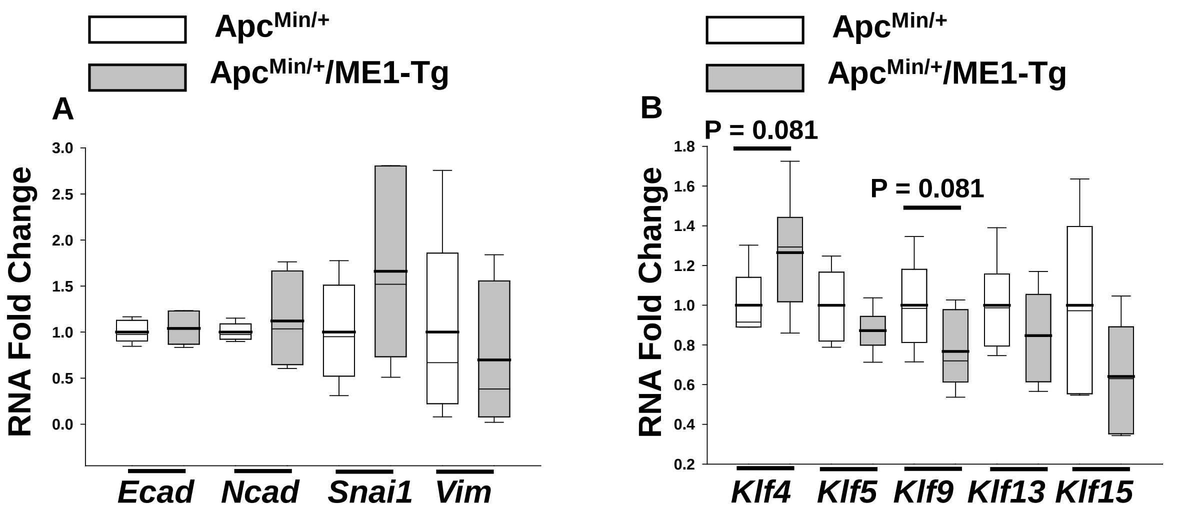

Supplementary Figure 4. Mouse intestine gene expression. (**A**) RNA fold changes for epithelial to mesenchymal transition (EMT)-associated genes in the jejunums from male $Apc^{Min/+}$ and $Apc^{Min/+}$/ME1-Tg mice (n=5-6/group). (**B**) RNA fold changes for *Klf/Sp* family members in jejunums from male $Apc^{Min/+}$ and $Apc^{Min/+}$/ME1-Tg mice (n=6/group). Boxes indicate the inter-quartile range of 25-75%, with mean (thick line) and median (thin line); whiskers: 10th and 90th percentiles. Student's *t*-tests were used to examine for differences between groups (none found). Tendencies for differences are indicated (0.1>P>0.05).

Supplementary Figure 5

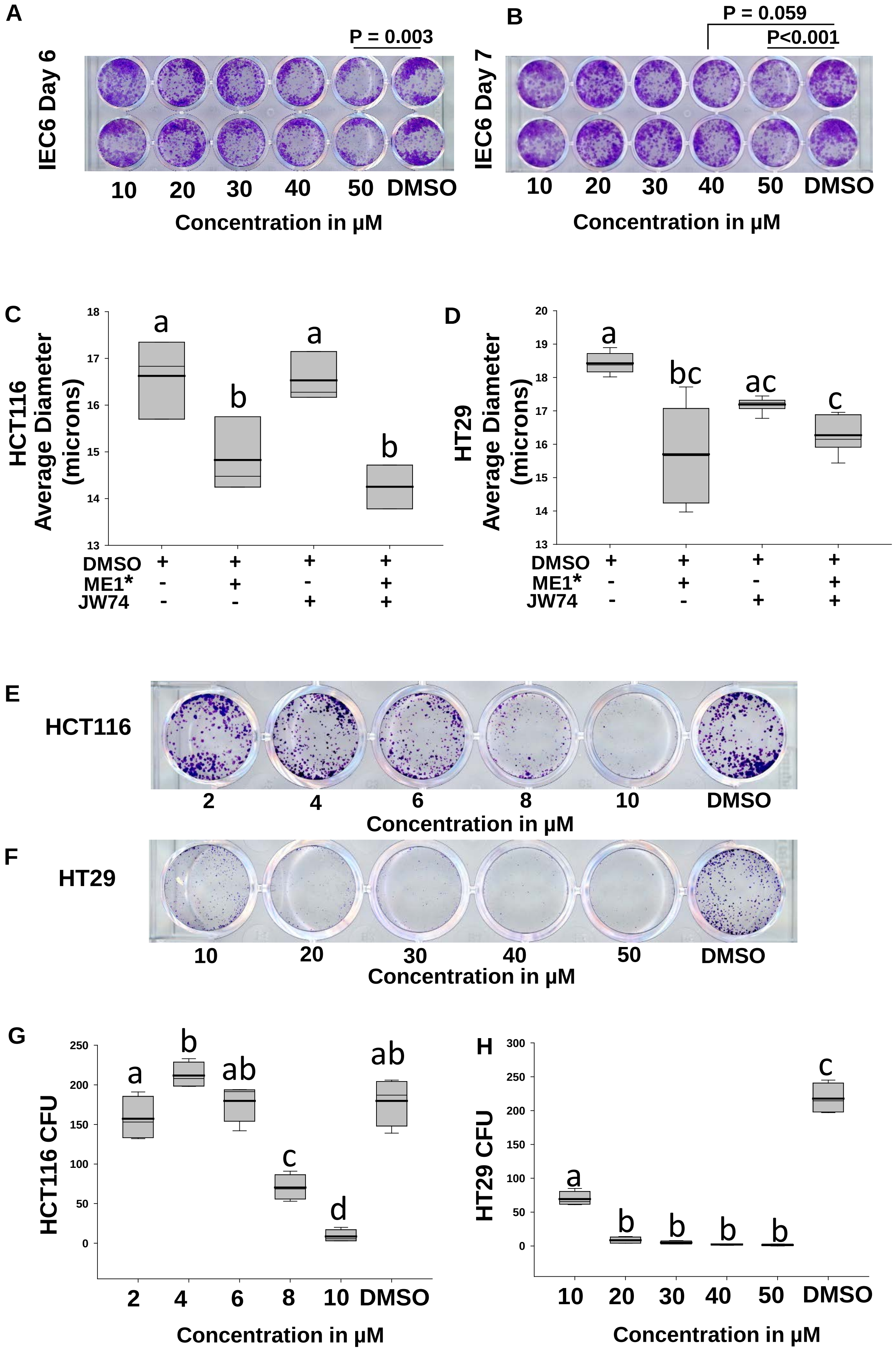

Supplementary Figure 5. (**A, B**) Clonogenic assay of IEC6 cells (A: after six days; B: after 7 days). (**C**, **D**) Quantitation of cell diameters after treatment with 50uM ME1*, 15 uM JW74, 50uM ME1* plus 15uM JW74, or vehicle (DMSO). Twenty thousand cells were plated in each well; after 24 h, cells were treated with inhibitors, and after 72 h of treatment were sized (n=3-6 wells/treatment group, 2 independent experiments). (**E**) HCT116 colonies in clonogenic assay. Cells were plated (1000 cells/well) and after 24 h were treated with varying doses of ME1* or vehicle (DMSO), incubated for six days, followed by crystal violet staining. (**F**) HT29 colonies in clonogenic assay. Cells were plated (1000 cells/well) and after 24 h were treated with various doses of ME1* or vehicle (DMSO), incubated for six days, followed by crystal violet staining. (**G, H**) Quantitation of HCT116 and HT29 colony forming units (CFU) (n=4 well/treatment dose). Boxes indicate the inter-quartile range of 25-75% with mean (thick line) and median (thin line); whiskers: 10th and 90th percentiles. One way ANOVA was used to compare differences between treatment groups. Different lowercase letters (a, b) designate groups significantly different from each other ($P<0.05$); bars sharing the same letter are not significantly different from each other.

Supplementary Figure 6

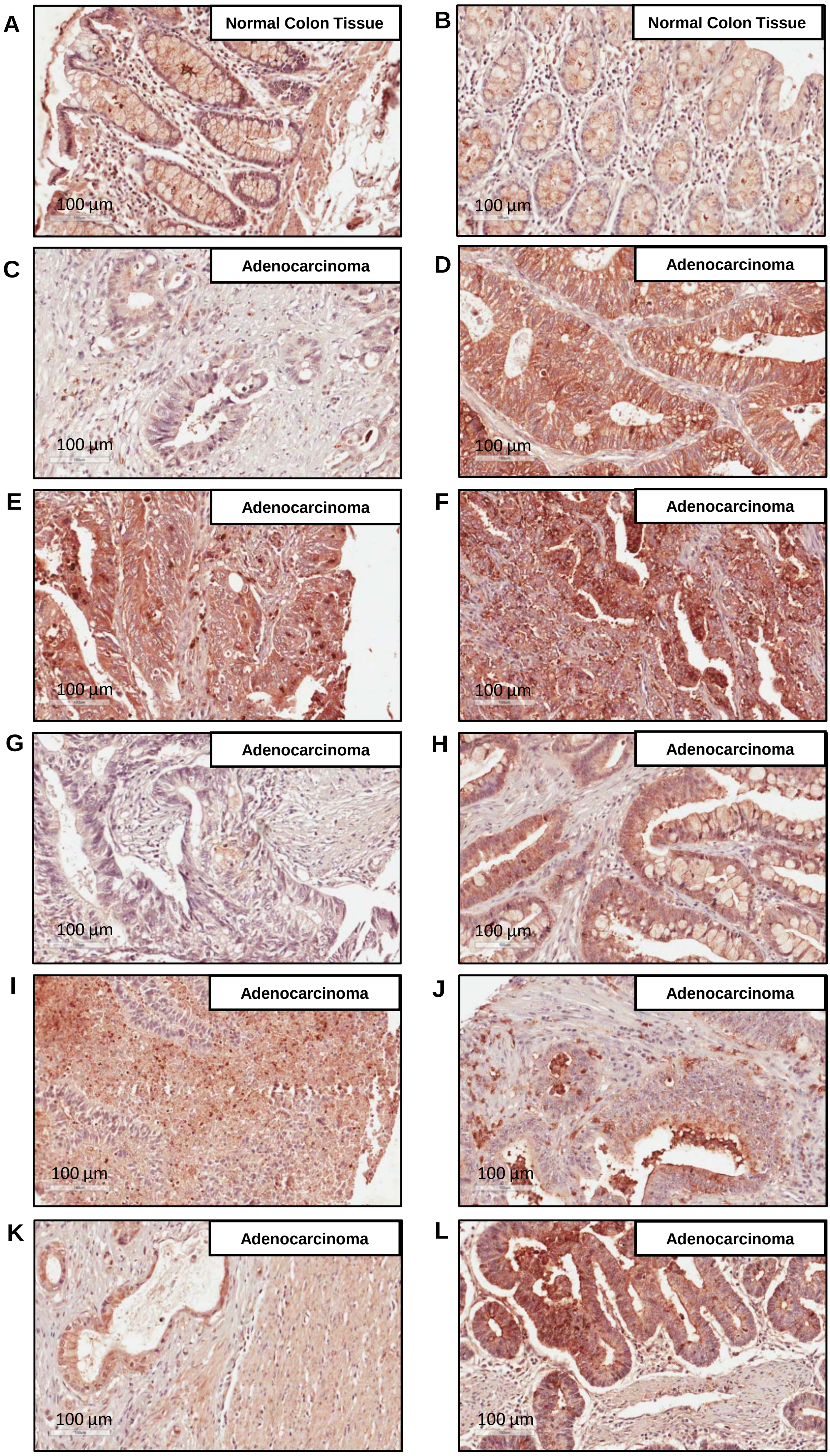

Supplementary Figure 6. ME1-IHC of normal human colon and human colon carcinoma tissues. (**A, B**) Normal colon tissue. (**C**) Stage I, adenocarcinoma. (**D**) Stage II, adenocarcinoma. (**E**) Stage III, adenocarcinoma. (**F**) Stage III, adenocarcinoma. (**G**) Stage IV, adenocarcinoma. (**H**) Stage II, mucinous adenocarcinoma. (**I**) Stage III, adenocarcinoma (smooth muscle and necrotic tissue). (**J**) Stage III, adenocarcinoma (chronic inflammation of smooth muscle). (**K**) Stage II, adenocarcinoma (smooth muscle). (**L**) Stage II, adenocarcinoma. (scale bars=100 um).

Supplementary Figure 7

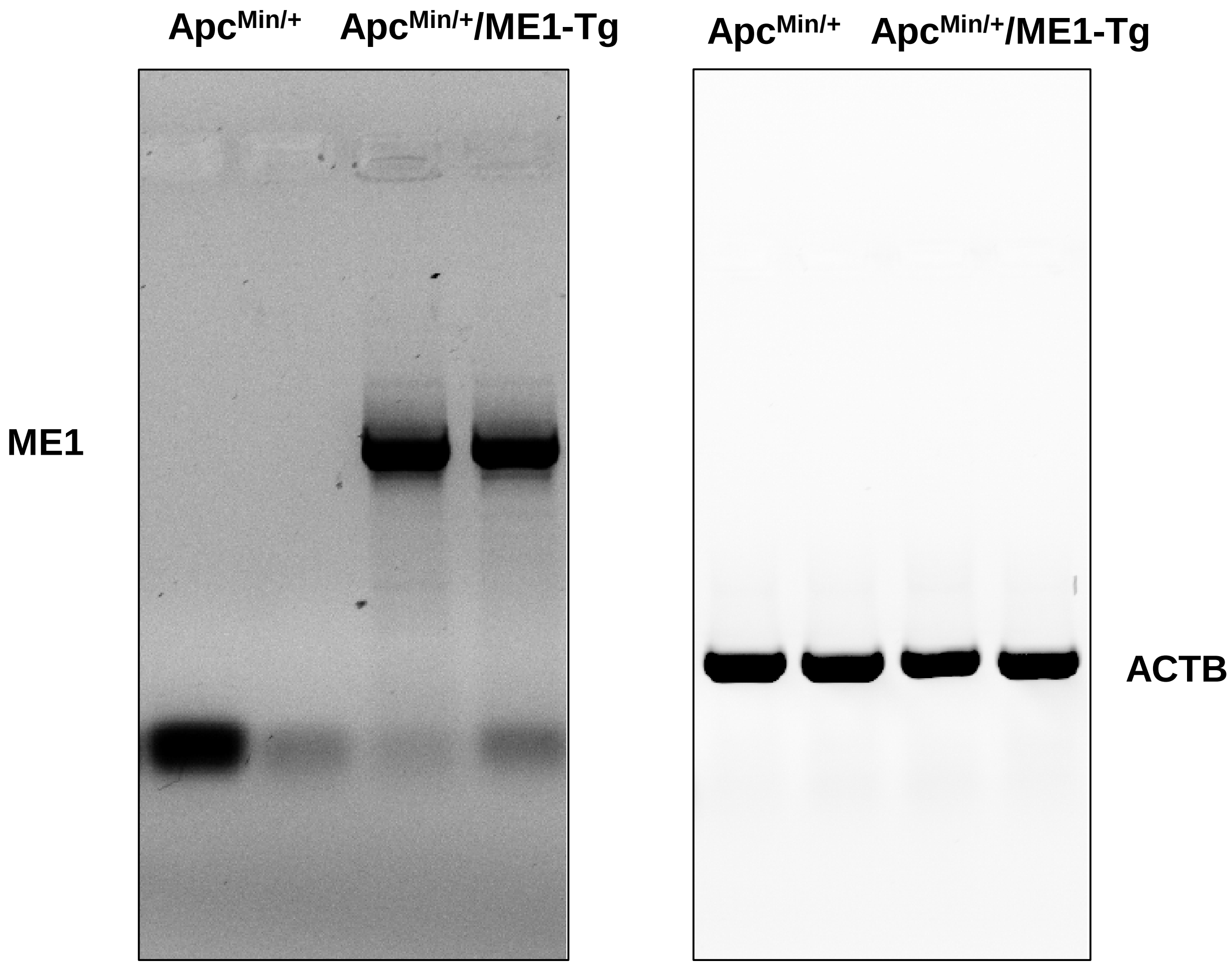

Supplementary Figure 7. Full-length gels for Figure 1C.
